# Supplementary material for: Bielectron vortices in two-dimensional Dirac semimetals
Source: Nat Commun. 2017 Oct 12;8:897. doi: 10.1038/s41467-017-00949-y (PMC5638912; doi:10.1038/s41467-017-00949-y)
Supplement: Supplementary file 1 — Supplementary Information [file 41467_2017_949_MOESM1_ESM.pdf]

SUPPLEMENTARY INFORMATION

Supplementary Note 1: On an image potential

As we already mentioned in the main body of this work, the presence of a metallic gate (leading to the appearance of image charges) suggests an interaction potential with a dipole-like ($1/r^3$) asymptotic decay. Let us consider the two dimensional Coulomb potential, with a regularization r_0 and dimensionless strength parameter γ, in a gated structure

$$U_I(r) = \gamma \hbar v_{\mathrm{F}} \left(\frac{1}{\sqrt{r_0^2 + r^2}} - \frac{1}{\sqrt{4s^2 + r^2}} \right), \tag{S1}$$

where s is the separation from the semimetal to the metallic back-gate. One numerical scheme to find the critical condition for two electrons interacting via Eq. (S1) to bind at some potential strength γ, is to expand the wavefunction component $\phi_2(r)$ as a Fourier-Bessel series: $\phi_2(r) = \sum_{j=1}^{\infty} a_j J_m(x_j r)$, where x_j are roots of the Bessel function of the first kind, $J_m(x)$. Evaluating the consequent matrix elements and solving the resulting secular equation numerically, leads to the desired values of $\gamma = \gamma_{n,|m|}(r_0/s)$, which correspond to two-particle pair bound states with quantum numbers n and $|m|$, and is a function of the dimensionless ratio r_0/s. Notably, the short range cutoff should be of the order of the carbon-carbon spacing $r_0 \simeq 0.142$ nm, whilst the graphene to back-gate separation can be in the range $s \sim 10$ nm to $s \sim 100$ nm. In this regime, one finds the following typical results for the critical parameter $\alpha_c(r_0/s) = \gamma_{0,1}(r_0/s)$, explicitly: $\alpha_c(r_0/s = 10^{-2}) \simeq 2.78$, $\alpha_c(10^{-3}) \simeq 2.48$, and $\alpha_c(10^{-4}) \simeq 2.37$. Thus, α_c is ordinarily just above the value of the unstrained graphene fine structure constant $\alpha \simeq 2.19/\kappa$, but below the fine structure constants of silicene ($\alpha \simeq 4.06/\kappa$) and germanene ($\alpha \simeq 4.13/\kappa$).

Supplementary Note 2: On a screened potential

In structures of 2D Dirac materials without a gate, screening can be seen to be the mechanism determining the criticality of the system. The Thomas-Fermi statically screened two dimensional Coulomb potential $U(q) = 2\pi\alpha\hbar v_{\mathrm{F}}(q + q_{\mathrm{TF}})^{-1}$ can be approximated by [1, 2]

$$U_S(r) = \gamma \hbar v_{\mathrm{F}} \frac{1}{\sqrt{r_0^2 + r^2}} \frac{1}{(1 + q_{\mathrm{TF}} r)^2}, \tag{S2}$$

which has a regularization parameter r_0, a Thomas Fermi wavevector q_{TF} and dimensionless strength γ. The critical strength requirement to sustain bound vortex pairs is a function of the dimensionless product $q_{\mathrm{TF}} r_0$, namely $\alpha_c = \alpha_c(q_{\mathrm{TF}} r_0)$. In direct comparison to the results with the image potential given above, we obtain $\alpha_c(q_{\mathrm{TF}} r_0 = 10^{-2}) \simeq 2.68$, $\alpha_c(10^{-3}) \simeq 2.44$, $\alpha_c(10^{-4}) \simeq 2.35$. Notably, static screening is well-known to be an overestimate compared to dynamical screening, such that the true α_c will be close to the value of the unstrained graphene fine structure constant $\alpha \simeq 2.19/\kappa$. Therefore, screening effects are important as they can lead to the disappearance of vortices at higher particle densities n, as follows from the relation $q_{\mathrm{TF}} = e^2\sqrt{4\pi g n}/\hbar v_{\mathrm{F}} \kappa$, where g is a factor introduced to count possible spin and valley degeneracies [3]. The effect of temperature on the Thomas-Fermi wavevector is discussed below.

Supplementary Note 3: On Thomas-Fermi screening at nonzero temperatures

Let us consider a gapless 2D Dirac material with charge carrier spectrum $E = \hbar v_{\mathrm{F}} |\mathbf{k}|$ and density of states $\rho(E) = gE/(2\pi\hbar^2 v_{\mathrm{F}}^2)$, where g accounts for any degeneracies in the system. The particle density at some temperature T is given by

$$n(\mu, \beta) = \frac{-g}{2\pi} \frac{1}{(\hbar v_{\mathrm{F}} \beta)^2} \mathrm{Li}_2\left(-e^{\beta\mu}\right), \tag{S3}$$

where μ is the chemical potential, $\beta = 1/k_B T$ and with the polylogarithm function

$$\mathrm{Li}_n\left(z\right) = \sum_{k=1}^{\infty} \frac{z^k}{k^n}. \tag{S4}$$

In the limit of zero temperature, one obtains $n = gE_F^2/(4\pi\hbar^2 v_F^2)$, where the Fermi energy $E_F = \mu(T = 0)$. Furthermore, it follows from Eq. (S3) that at finite temperature

$$\frac{\partial n}{\partial \mu} = \frac{g}{2\pi} \frac{1}{(\hbar v_F)^2} \frac{\ln\left(1 + e^{\beta\mu}\right)}{\beta}, \tag{S5}$$

which tends to $\partial n/\partial\mu = gE_F/(2\pi\hbar^2 v_F^2)$ in the limit of vanishing temperature. This quantity $\partial n/\partial\mu$ is important, since the Thomas-Fermi screening wavevector in 2D is given by $q_{TF} = (2\pi e^2/\kappa)\, \partial n/\partial\mu$ [4]. At zero temperature, it can be readily seen that the screening wavevector $q_{TF}(T = 0) = e^2\sqrt{4\pi g n}/\hbar v_F \kappa$ increases with the square root of the particle density. This implies that above a critical particle density the system will be in a supercritical state, and as such unable to support bielectron vortices. In Supplementary Figure 1 we plot the screening wavevector q_{TF} as a function of particle density n for a various temperatures. Most notably, the effect of a finite temperature is to slightly reduce the screening wavevector for a given number density, such that the formation of bielectron vortices is further preserved compared to the zero temperature scenario.

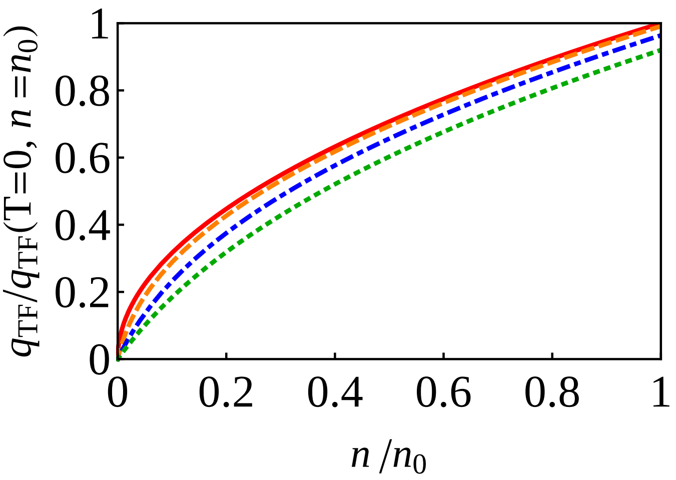

Supplementary Figure 1: **The relationship between screening wavevector and particle density for a 2D Dirac-Weyl system** A plot of the Thomas-Fermi wavevector q_{TF} as a function of particle density n, for the temperatures $T = 0$K (solid red line), $T = 100$K (dashed orange line), $T = 200$K (dot-dashed blue line) and $T = 300$K (dotted green line). The reference particle density $n_0 = 10^{12}\text{cm}^{-2}$.

Supplementary References

[1] C. Tanguy, *Counting the number of bound states of two-dimensional screened Coulomb potentials: a semiclassical approach*, preprint at https://arxiv.org/abs/cond-mat/0106184 (2001).

[2] D. G. W. Parfitt and M. E. Portnoi, *Two-dimensional exciton revisited*, Physica E **17**, 212-214 (2003).

[3] S. Das Sarma, S. Adam, E. H. Hwang and E. Rossi, *Electronic transport in two-dimensional graphene*, Rev. Mod. Phys. **83**, 407-470 (2011).

[4] H. Haug and S. Koch, *Quantum Theory of Optical and Electronic Properties of Semiconductors*, 4th ed. (World Scientific Publishing, Singapore, 2004).
